# Evaluation of SUVlean consistency in FDG and PSMA PET/MR with Dixon-, James-, and Janma-based lean body mass correction

**DOI:** 10.1186/s40658-021-00363-w

**Published:** 2021-02-17

**Authors:** Jun Zhao, Qiaoyi Xue, Xing Chen, Zhiwen You, Zhe Wang, Jianmin Yuan, Hui Liu, Lingzhi Hu

**Affiliations:** 1 Department of Nuclear Medicine, Shanghai East Hospital, Tongji University School of Medicine, Shanghai, China; 2 Central Research Institute, United Imaging Healthcare Group, Shanghai, China

**Keywords:** SUV, Lean body mass, PET/MR

## Abstract

**Purpose:**

To systematically evaluate the consistency of various standardized uptake value (SUV) lean body mass (LBM) normalization methods in a clinical positron emission tomography/magnetic resonance imaging (PET/MR) setting.

**Methods:**

SUV of brain, liver, prostate, parotid, blood, and muscle were measured in 90 ^18^F-FDG and 28 ^18^F-PSMA PET/MR scans and corrected for LBM using the James, Janma (short for Janmahasatian), and Dixon approaches. The prospective study was performed from December 2018 to August 2020 at Shanghai East Hospital. Forty dual energy X-ray absorptiometry (DXA) measurements of non-fat mass were used as the reference standard. Agreement between different LBM methods was assessed by linear regression and Bland-Altman statistics. SUV’s dependency on BMI was evaluated by means of linear regression and Pearson correlation.

**Results:**

Compared to DXA, the Dixon approach presented the least bias in LBM/weight% than James and Janma models (bias 0.4±7.3%, − 8.0±9.4%, and − 3.3±8.3% respectively). SUV normalized by body weight (SUVbw) was positively correlated with body mass index (BMI) for both FDG (e.g., liver: *r* = 0.45, *p* < 0.001) and PSMA scans (*r* = 0.20, *p* = 0.31), while SUV normalized by lean body mass (SUVlean) revealed a decreased dependency on BMI (*r* = 0.22, 0.08, 0.14, *p* = 0.04, 0.46, 0.18 for Dixon, James, and Janma models, respectively). The liver SUVbw of obese/overweight patients was significantly larger (*p* < 0.001) than that of normal patients, whereas the bias was mostly eliminated in SUVlean. One-way ANOVA showed significant difference (*p* < 0.001) between SUVlean in major organs measured using Dixon method vs James and Janma models.

**Conclusion:**

Significant systematic variation was found using different approaches to calculate SUVlean. A consistent correction method should be applied for serial PET/MR scans. The Dixon method provides the most accurate measure of LBM, yielding the least bias of all approaches when compared to DXA.

**Supplementary Information:**

The online version contains supplementary material available at 10.1186/s40658-021-00363-w.

## Introduction

The unique benefits of integrated positron emission tomography/magnetic resonance imaging (PET/MR), including comprehensive contrast mechanisms and seamless fusion of morphology and function, are driving adoption and exploration in both the clinical and research domains. Because of the reduced radiation dose of PET/MR versus PET/CT, and the increasing utilization of non-FDG (fluorodeoxyglucose) tracers, such as PSMA (prostate-specific membrane antigen) and DOTATATE [1, 2], serial PET/MR scans are becoming more desired for re-staging and treatment response evaluation for oncological patients. Patients might present dramatic physiological variation in terms of body weight throughout the course of treatment, requiring serial PET scans to maintain high standards for consistent and accurate quantitation [3].

In PET studies, standardized uptake value (SUV) is the most widely used semiquantitative measurement of radiotracer uptake, essential for diagnosis and treatment response assessment. SUV is defined as the radioactivity in a region of interest (ROI) normalized to the total injected dose and body weight of the patient [4]. Although SUV normalized to body weight (SUVbw) is the most popular metric in today’s clinical setting, Zasadny and Wahl found that it is highly dependent on patient weight and body fat content [5]. A potential cause of inconsistency is that white adipose tissue minimally uptakes radiotracer but contributes to overall body weight. SUVbw is occasionally overestimated (especially for obese subjects) and can lead to systematic bias for serial scans of patients with multiple follow-ups throughout the course of treatment. Many studies have investigated methods to improve normalization factors of SUV to account for more consistent quantitation across a wide range of body mass indices (for example, BMI = weight/height^2^). The most widely adopted approach is to use lean body mass (LBM) instead of body weight to offset the systematic bias caused by white adipose tissue [5]. This corrected SUV is often referred to as SUVlean or SUL. SUVlean has been recommended by PERCIST and has been widely accepted in clinical and research studies [6, 7].

Over the past decades, various predictive models have been established to estimate LBM, taking factors such as body weight, height, sex, and/or age into account. Some of these models have been translated into PET imaging to calculate SUVlean in the clinical setting [5, 8–10]. Among them, James equation [11] is the most widely used model for SUV correction and has been implemented in a variety of commercially available PET/CT and PET/MR scanners. However, a recent study has shown that James equation might be prone to significant inaccuracy when a patient’s BMI exceeds a critical value (approximately 43 for men and 37 for women) [12]. An improved model proposed by Janmahasatian et al. [13] was adopted in some recent studies [12, 14] to improve the SUV consistency for patients with high BMI. However, even though this model-based LBM estimation was derived from extensive clinical data containing a large patient cohort, there are still concerns that the predictive formulae may cause substantial errors at the individual level. For instance, two patients with the same weight and height would exhibit identical LBM values but may present significantly different body fat composition.

New LBM estimation approaches based on direct measurement using CT or MR from PET/CT and PET/MR [15–18] are believed to be more reliable than model-based LBM methods [14, 19, 20]. For PET/MR imaging, the current state of the art MR-based attenuation correction (MRAC) utilizes a Dixon sequence to generate water/fat images [21] and such data could be readily utilized to obtain personalized calculation of LBM. The Dixon approach was recently suggested and validated in a pilot study, where Jochimsen et al. reported an initial attempt to normalize SUV with the Dixon-based water/fat fraction [22]. Good reproducibility and robust LBM measurement using the Dixon method was later reported by Rausch et al. [23].

Furthermore, it would be challenging to conduct an evaluation of SUVlean accuracy without validating these methods for LBM calculation against the current clinical reference standards [24]. One approach for validating and comparing different SUV normalization models is to utilize reference standard measurements of LBM [25] using well-established technology [19, 26]. There are many ways to measure body fat and its distribution [27, 28]. For instance, dual energy X-ray absorptiometry (DXA) utilizes different attenuation coefficient of fat and soft tissue to obtain a patient-specific fat fraction [27, 29]. DXA has been reported to be more accurate than density-based methods and features good repeatability of regional fat fraction obtained by cropping the projected 2D coronal image [30].

Although a few recent studies have reported good reproducibility of the Dixon method [23] and good agreement between Dixon and DXA measurements of body fat [17, 31, 32], the robustness of PET SUV corrected by the Dixon method has not been well evaluated. In addition, because of the broad selection of methods to calculate LBM for SUVlean, there is an immediate need for comparative evaluation of the consistency and limitation across these methods for SUVlean calculations. The purpose of the present work is to systematically evaluate the accuracy of different LBM estimation methods, using DXA as a reference standard, and to investigate the consistency of various SUVlean calculations in a clinical setting. SUVlean measurements derived from Dixon images, as well as with James and Janma (short for Janmahasatian) LBM models, were compared in two patient cohorts of ^18^F-FDG and ^18^F-PSMA PET/MR studies, respectively. To our knowledge, this is the first study to evaluate these three SUVlean methods in both an FDG and PSMA PET/MR cohort.

## Materials and methods

### Patient population

**Fig. 1:**
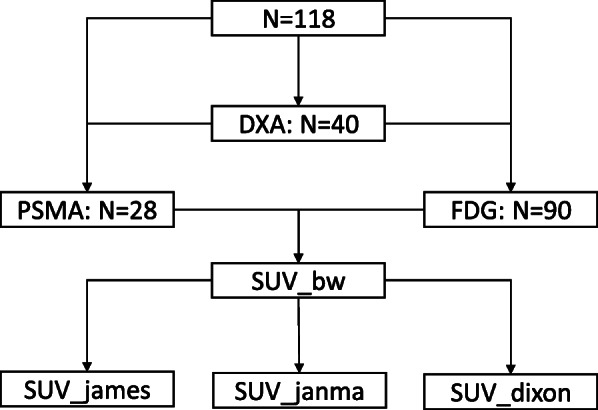
A flow chart of study design and patient enrollment

Patients (*N* = 118) were recruited for clinical PET/MR scans from December 2018 to August 2020 at Shanghai East Hospital for suspected or known malignancies. Among them, 90 underwent ^18^F-FDG PET/MR scans and 28 underwent ^18^F-PSMA PET/MR scans. Forty out of 118 patients were enrolled in a same day DXA scan for body fat measurement. Patient weight ranged from 37 to 103 kg and BMI ranged from 14.53 to 32.45. Patients with metal implant and claustrophobia to MRI were excluded in this study. The study design is summarized in Fig. 1 and detailed information of the patients is provided in Table 1. The study protocol was reviewed and approved by institutional review board (IRB), and written informed consent was obtained from each patient.

**Table 1:** Characteristics of recruited patients

	FDG	PSMA	DXA
*N*	48F, 42M	28M	20F, 20M
Age	58 ± 13 (17~91)	70 ± 7 (56~89)	60 ± 12
Weight (kg)	63 ± 14.4 (37~103)	70.7 ± 8.4 (54~87)	64.7 ± 12.6 (43~94)
BMI	23.11 ± 4.54 (14.5~32.5)	24.47 ± 2.55 (18.7~28.3)	23.47 ± 3.5 (15~31)
Height (cm)	165 ± 7 (150~183)	170 ± 5 (162~180)	165 ± 7 (152~180)
Time (min)	72 ± 14	141 ± 18	/
Dose (mCi/kg)	9.6 ± 1.7%	12.1 ± 3.1%	/
Dose (MBq)	221 ± 50	314 ± 73	/

*BMI* body mass index, *FDG* fluorodeoxyglucose, *PSMA* prostate-specific membrane antigen

### PET/MR image acquisition

Whole-body PET/MR scans were performed on a hybrid PET/MR (uPMR 790, UIH, Shanghai, China), which consisted of a 3.0T MR and PET system with a transverse field of view of 60 cm and axial field of view of 32 cm. The PET system comprises 112 rings, each containing 700 15.5 × 2.76 × 2.76 mm^3^ LYSO crystals [33]. The PET system quality control was performed on a daily basis. All patients were requested to fast for at least 6 h before the injection of the radioactive tracer. For the FDG study, patients were injected with 221 ± 50 MBq (or 0.096 ± 0.017 mCi/kg) of ^18^F-FDG and rested in a quiet preparation room for about 1 h. For the PSMA study, patients were injected with 314 ± 73 MBq of ^18^F-PSMA-1007 and rested for about 2 h.

**Fig. 2:**
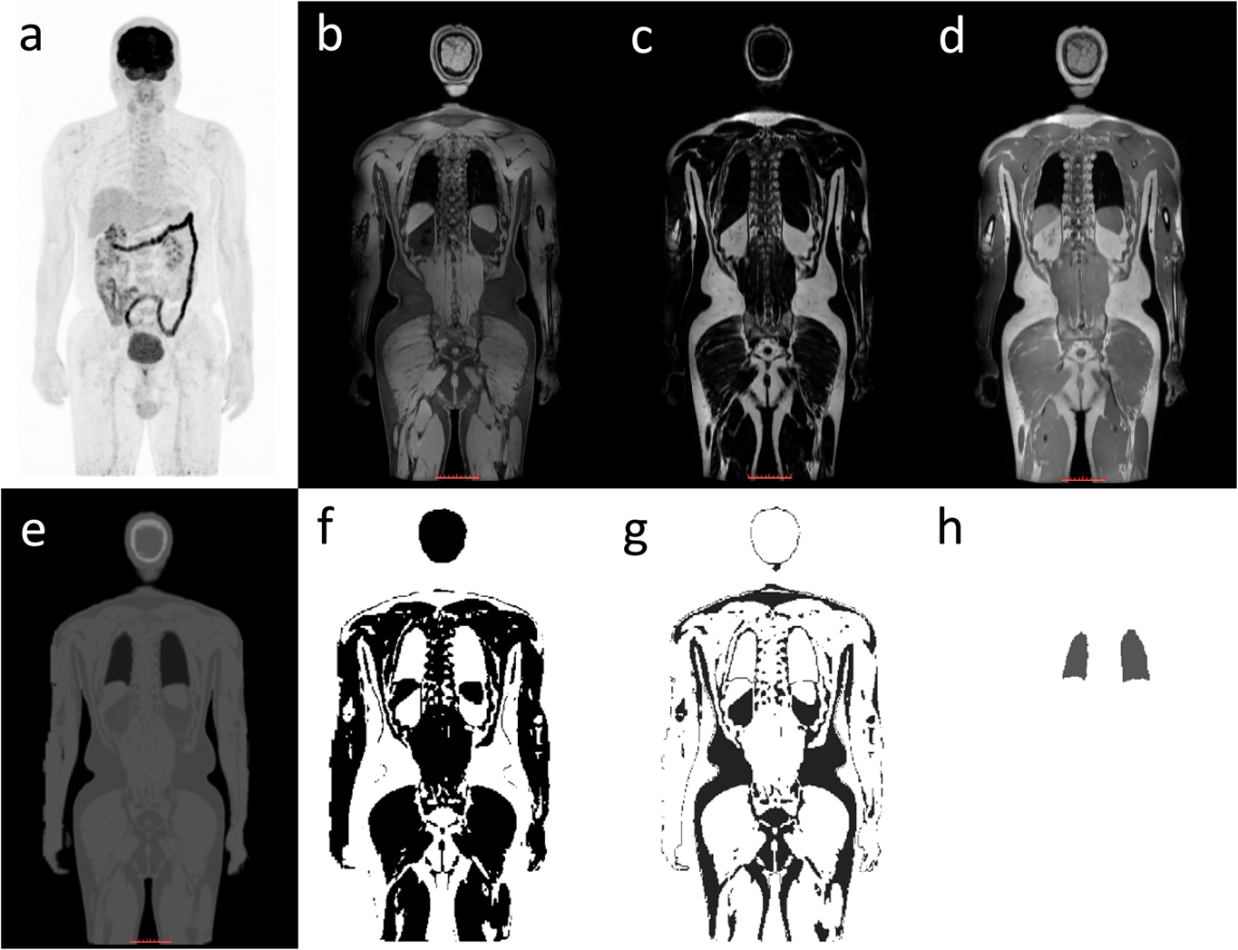
Representative images from a ^18^F-FDG PET/MR scan. **a** PET MIP, **b** water image, **c** fat image, **d** in-phase image, **e** MRAC map, **f** water segmented from MRAC, **g** fat segmented from MRAC, and **h** lung segmented from MRAC

**Fig. 3:**
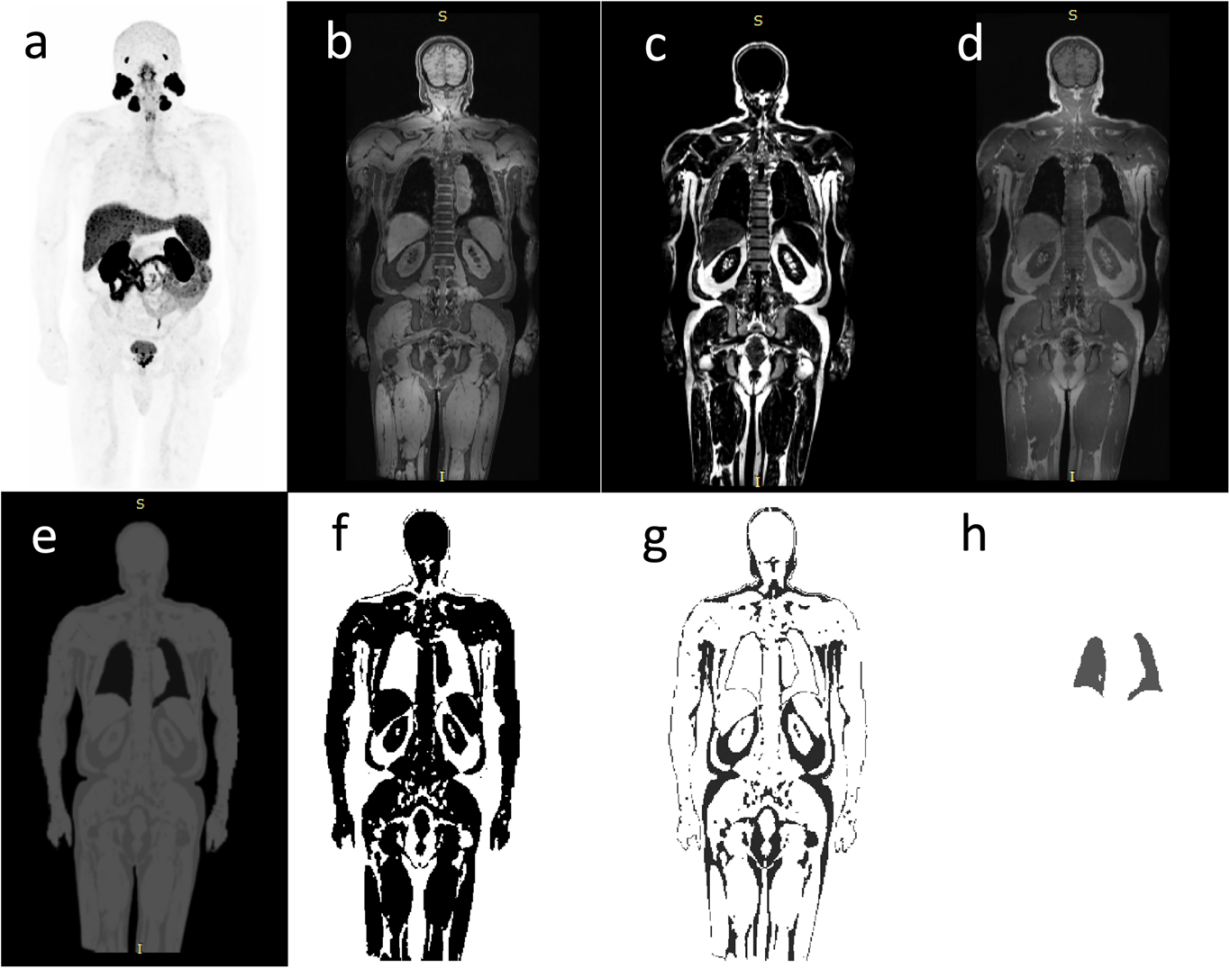
Representative images from a ^18^F-PSMA PET/MR scan. **a** PET MIP, **b** water image, **c** fat image, **d** in-phase image, **e** MRAC map, **f** water segmented from MRAC, **g** fat segmented from MRAC, and **h** lung segmented from MRAC

Images were acquired using the clinical PET/MR protocol at Shanghai East Hospital. During the PET scan, a 2-point Dixon-based water-fat separation imaging sequence was performed simultaneously using a 3D T1-weighted gradient echo sequence with compressed sensing (TE = 2.24 ms, TR = 4.91 ms, flip angle = 10, echo train length = 30, FOV = 549 × 384, matrix = 256 × 329, slice thickness = 2mm, slice spacing = 2mm, transverse plane). The PET/MR MRAC map was generated by segmenting the Dixon images into water, fat, lung, and air and assigning attenuation coefficients of 0.096, 0.08, 0.032, and 0 cm^−1^, respectively. Representative images of PET/MR FDG and PSMA are shown in Figs. 2 and 3 respectively.

### DXA image acquisition

DXA images were obtained using a dual-energy X-ray absorptiometry (DXA) (Lunar Prodigy, GE, Madison, USA) and the body composition was analyzed using vendor-provided software (enCORE, GE, Madison, USA). Since the PET/MR scan coverage was from skull to the upper thigh, the ROI of the DXA images were adjusted to match the axial coverage as the PET/MR image to calculate the total mass and fat mass.

### LBM calculation

The total volume of water, fat, lung, and air were obtained from the Dixon MRAC images by multiplying the total number of voxels by the voxel volume for each compartment. We first compared, using linear regression, the MRAC fat and water volumes with the mass derived from DXA analysis to assess the agreement between the two measurements. Population-based water and fat density were derived by using a linear fit of the MRAC volume versus the measured water and fat mass from DXA.

Model-based LBM were calculated using James equation and Janma equation respectively,

where:

1 $$ \mathrm{LBWJanma}=\left\{\begin{array}{c}\raisebox{1ex}{$9270\ast \mathrm{BW}$}\!\left/ \!\raisebox{-1ex}{$6680+216\ast \mathrm{BMI}$}\right.,\kern0.5em \mathrm{Men}\\ {}\raisebox{1ex}{$9270\ast \mathrm{BW}$}\!\left/ \!\raisebox{-1ex}{$8780+244\ast \mathrm{BMI}$}\right.,\mathrm{Women}\end{array}\right. $$

and

2 $$ \mathrm{LBWJames}=\left\{\begin{array}{c}1.1\ast \mathrm{BW}-128\ast {\left(\frac{\mathrm{BW}}{\mathrm{height}}\right)}^2,\mathrm{Men}\\ {}1.07\ast \mathrm{BW}-148\ast {\left(\frac{\mathrm{BW}}{\mathrm{height}}\right)}^2,\mathrm{Women}\end{array}\right. $$

where BW is the body weight and LBWjanma/james are the lean body mass calculated by Janma and James equation. The results were compared with the direct measurement result from DXA as the reference standard.

### PET reconstruction and SUV measurements

All PET reconstructions and image analysis were performed on the vendor-provided workstation (uPMR 790, UIH, Shanghai, China). The PET images were reconstructed using the ordered subset expectation maximization (OSEM) algorithm (FOV = 600 mm, iteration = 2, subsets = 20, Gaussian filter with FWHM = 4 mm, matrix size = 150). The radioactivity of the major organs was measured by drawing ROIs on the workstation. Specifically, the radioactivity of the liver was obtained by placing a 3-cm diameter spherical ROI in the right hepatic lobe avoiding major vessels/lesions according to the PERCIST criteria [6]. The radioactivity of the blood pool was obtained by placing a 1-cm diameter spherical ROI in the left ventricle, and the muscle radioactivity was obtained from the right thigh. Brain radioactivity in the FDG studies was obtained by thresholding out the whole brain using an in-house algorithm which was then subsequently validated by visual inspection. Prostate and parotid glands in the PSMA studies were detected by a thresholding tool available in the workstation software. SUVpeak and SUVmean of lymph node metastasis with avid PSMA uptake were measured by the same tool. SUVpeak was defined as the average value within a 1 cm^3^ volume around the SUVmax.

SUVbw was calculated by the default settings in the workstation as:

3 $$ \mathrm{SUVbw}=\frac{\mathrm{radioactivity}\ \mathrm{in}\ \mathrm{ROI}\left(\mathrm{kBq}/\mathrm{ml}\right)}{\mathrm{injected}\ \mathrm{dose}\left(\mathrm{MBq}\right)\ast \mathrm{decay}\ \mathrm{factor}/\mathrm{bodyweight}\left(\mathrm{kg}\right)} $$

where decay factor = exp(− 0.693*wait time/radionuclide half-life).

SUVlean calculated using Dixon, James, and Janma approaches were denoted as SUV_dixon, SUV_james, and SUV_janma, respectively.

SUV_dixon was calculated as:

4 $$ \mathrm{SUV}\_\mathrm{dixon}=\mathrm{SUVbw}\ast \frac{\mathrm{water}\ \mathrm{mass}}{\mathrm{water}\ \mathrm{mass}+\mathrm{fat}\ \mathrm{mass}+\mathrm{lung}\ \mathrm{mass}} $$

where water and fat mass were derived from the Dixon MRAC images.

SUV_james was calculated as:

5 $$ \mathrm{SUV}\_\mathrm{james}\kern0.5em =\kern0.5em \mathrm{SUVbw}\kern0.5em \ast \kern0.5em \mathrm{LBWjames}/\mathrm{bodyweight}. $$

Finally, SUV_janma was calculated as:

6 $$ \mathrm{SUV}\_\mathrm{janma}\kern0.5em =\kern0.5em \mathrm{SUVbw}\kern0.5em \ast \kern0.5em \mathrm{LBWjanma}/\mathrm{bodyweight}. $$

### Statistical analysis

LBM calculated from Dixon, James, and Janma approaches were compared with the reference standard DXA calculations. Pearson correlation, paired *t* tests, and Bland-Altman analysis were used to evaluate the measurement accuracy of different LBM methods. Scatter plots with linear regression were used to determine the relationship between SUVlean and BMI (supplementary material). Linear regression and Pearson correlation were used to assess the dependency of SUVbw and SUVlean on BMI. One-way ANOVA was used to check agreement between different SUV normalization methods. A Kolmogorov-Smirnov test (KS test) was used to check the normal assumption of the data before performing *t* tests and ANOVA. All statistical analysis was performed in Matlab 2018b (MathWorks, Natick, MA, USA) and GraphPad Prism 8.0.2 (GraphPad Software, CA, USA).

## Results

The KS test showed that the LBM fraction calculated using Dixon, James, Janma, and DXA as well as the BMI and SUV values all followed normal distribution.

### DXA vs Dixon

**Fig. 4:**
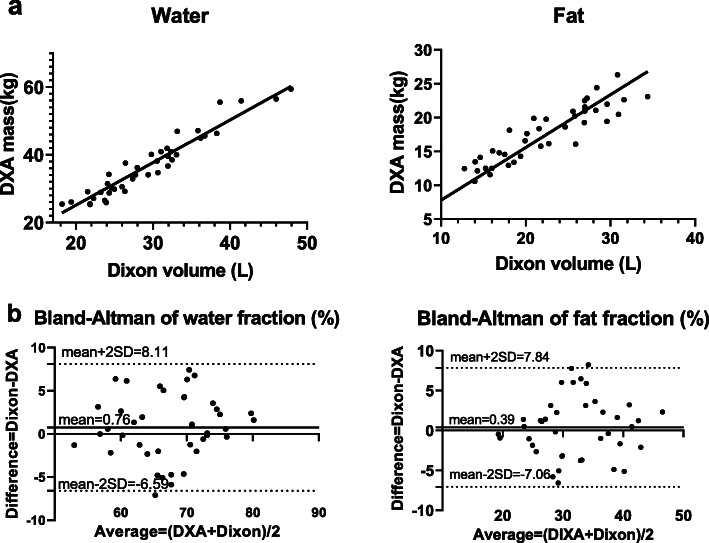
**a** Linear regression of volume measured by Dixon MRI vs. mass measured by DXA from head to upper thigh. The solid line represents the linear fit of the data. **b** Bland-Altman plot showing agreement between fat mass/bodyweight of the DXA and Dixon measurements, and water mass/bodyweight of the DXA and Dixon measurements

The average body weight for the 20 female subjects was 58.65 ± 8.70 kg and the average body weight for the 20 male subjects was 70.72 ± 12.93kg. The average BMI for women was 22.57 ± 3.18 and average BMI for men was 24.37 ± 3.51. The mean non-fat mass of the head-to-thigh region obtained in DXA was 30.14 ± 3.90 kg for women and 43.47 ± 7.86 kg for men. The corresponding total water volume obtained by the Dixon technique was 244.53 ± 3.21 L for women and 344.92 ± 5.67 L for men. The slope from the linear regression was 0.78 and 1. 26 for fat and water, with *r*^2^ = 0.849 and 0.915 respectively, suggesting excellent agreement between the measurements of DXA and Dixon (Fig. 4a). To convert MRI-measured volumes to weight in the recruited patient population, lung volumes were taken into account for multi-parameter linear regression, yielding coefficients (densities) of 0.79, 1.23, and 0.20 for fat, water and lung respectively. Using the derived tissue density, the fat and water mass was determined as 17.38 ± 5.05 kg and 33.50 ± 6.84 kg from the Dixon images. The Bland-Altman plot of fat fraction and water mass fraction are shown in Fig. 4b. The bias was 0.76 and 0.39 for water fraction and fat fraction respectively, and all points, except for one, fell into a confidence interval of two standard deviations.

### LBM fraction

The measured LBM fraction using DXA were 63.01 ± 3.91%, 73.37 ± 3.71%, and 68.19 ± 6.43% for females, males, and all patients respectively. As summarized in Table 2, LBM fraction calculated using Dixon, James model, and Janma model were measured to be 62.68 ± 5.74%, 73.59 ± 4.82%, and 65.08 ± 3.67% for female patients; 71.22 ± 4.59%, 78.80 ± 4.74%, and 77.95 ± 5.45% for male patients; and 66.95 ± 6.71%, 76.199 ± 5.406%, and 71.56 ± 7.97% for all patients respectively. Good correlation was found between Dixon LBM and DXA LBM in all groups. Paired *t* tests showed that LBM fraction calculated by James and Janma were significantly different from the DXA results (*p* < 0.05) in all groups, whereas Dixon LBM was not significantly different from DXA in the female group and the all-gender group. Dixon had the smallest bias and strongest correlation with DXA, suggesting that the Dixon method offers the most accurate measurement of LBM. Both the James and Janma model tend to overestimate LBM for all patients. James model yielded the largest bias when compared with the DXA measurement, especially for female, where LBM was overestimated by 10%.

**Table 2:** LBM calculated using the Dixon, James, and Janma models compared with DXA as the reference standard

(Unit: 100%)		Mean + SD	Bias ± 2SD	Paired *t* test	Correlation with DXA
Female, *N* = 20, 63.01±3.91	Dixon	63.04 ± 5.73	− 1.14 ± 7.84	0.217	0.718
	James	73.59 ± 4.82	− 10.59 ± 6.79	< 0.001	0.707
	Janma	65.08 ± 3.67	− 2.07 ± 5.66	0.005	0.721
Male, *N* = 20, 73.37 ± 3.71	Dixon	71.50 ± 4.56	2.02 ± 5.27	0.003	0.809
	James	78.80 ± 4.74	− 5.44 ± 8.95	< 0.001	0.446
	Janma	77.95 ± 5.45	− 4.59 ± 9.75	< 0.001	0.468
All, *N* = 40, 68.19 ± 6.43	Dixon	67.27 ± 6.67	0.44 ± 7.30	0.460	0.853
	James	76.20 ± 5.41	− 8.01 ± 9.36	< 0.001	0.694
	Janma	71.51 ± 7.97	− 3.33 ± 8.26	< 0.001	0.850

*LBM* lean body mass, *DXA* dual energy X-ray absorptiometry, *SD* standard deviation

### SUV in FDG study

Linear regression between FDG SUVbw and SUVlean calculated using different approaches of LBM are plotted in supplementary material and the quantitative statistical results are summarized in Table 3. Pearson correlation between SUVbw and BMI was greater than that of SUVlean in all tissues and was found to be significantly correlated for all tissues (*p* < 0.01) except for brain SUVmax. The dependency on BMI was eliminated when using either Dixon, James, or Janma-based SUVlean calculations resulting in non-significant correlation for most cases. Reduced dependence on BMI is also demonstrated by the reduced slope of linear regression shown in the scatter plot (supplementary material).

**Table 3:** Fitting results and dependent analysis of SUV vs. BMI for the FDG study (*n* = 90)

			Mean ± SD	CV	Slope with	Correlation with BMI	Significance of correlation with BMI (*p*)	ANOVA (*p*)
Brain	Max	bw	20.69 ± 8.86	0.428	0.417	0.173	0.103	*p* < 0.001
		Dixon	13.39 ± 5.77	0.430	0.132	0.067	0.531	0.052
		James	15.70 ± 6.59	0.420	0.094	0.032	0.767	
		Janma	14.68 ± 6.28	0.427	0.128	0.061	0.567	
	Mean	bw	5.72 ± 1.46	0.255	0.115	0.317	0.002	*p* < 0.001
		Dixon	3.71 ± 0.97	0.260	0.037	0.143	0.178	*p* < 0.001
		James	4.34 ±1.04	0.239	0.024	0.078	0.465	
		Janma	4.05 ± 1.02	0.251	0.033	0.124	0.245	
Liver	Max	bw	1.89 ± 0.37	0.196	0.043	0.536	*p* < 0.001	*p* < 0.001
		Dixon	1.22 ± 0.23	0.187	0.015	0.316	0.002	*p* < 0.001
		James	1.43 ± 0.24	0.165	0.010	0.211	0.046	
		Janma	1.34 ± 0.25	0.185	0.012	0.254	0.016	
	Mean	bw	1.59 ± 0.28	0.179	0.029	0.454	*p* < 0.001	*p* < 0.001
		Dixon	1.03 ± 0.17	0.165	0.008	0.217	0.04	*p* < 0.001
		James	1.21 ± 0.19	0.159	0.003	0.079	0.459	
		Janma	1.13 ± 0.20	0.176	0.005	0.143	0.180	
Blood	Max	bw	1.02 ± 0.20	0.192	0.019	0.402	*p* < 0.001	*p* < 0.001
		Dixon	0.66 ± 0.11	0.162	0.005	0.189	0.074	*p* < 0.001
		James	0.77 ± 0.13	0.171	0.002	0.056	0.603	
		Janma	0.72 ± 0.13	0.186	0.004	0.114	0.283	
	Mean	bw	0.90 ± 0.19	0.207	0.014	0.306	0.003	*p* < 0.001
		Dixon	0.58 ± 0.10	0.176	0.003	0.093	0.382	*p* < 0.001
		James	0.69 ± 0.13	0.189	0.000	− 0.024	0.822	
		Janma	0.64 ± 0.13	0.197	0.001	0.039	0.714	
Muscle	Max	bw	0.65 ± 0.16	0.253	0.016	0.426	*p* < 0.001	*p* < 0.001
		Dixon	0.42 ± 0.09	0.220	0.006	0.280	0.008	*p* < 0.001
		James	0.49 ± 0.11	0.220	0.005	0.189	0.074	
		Janma	0.46 ± 0.10	0.227	0.005	0.232	0.028	
	Mean	bw	0.54 ± 0.13	0.237	0.010	0.348	*p* < 0.001	*p* < 0.001
		Dixon	0.35 ± 0.07	0.196	0.003	0.182	0.086	*p* < 0.001
		James	0.41 ± 0.08	0.207	0.002	0.077	0.468	
		Janma	0.38 ± 0.08	0.211	0.002	0.126	0.237	

*SUV* standardized uptake value, *BMI* body mass index, *CV* coefficient of variation, *DXA* dual energy X-ray absorptiometry, *SD* standard deviation, *ANOVA* analysis of variance

Coefficient of variance (CV) for all SUVlean measurements was smaller than that of SUVbw measurements, suggesting a smaller variance among patients. CV of SUV_dixon, SUV_james, and SUV_janma measurements were comparable. The one-way ANOVA among the 4 SUVs methods showed that SUVbw was significantly different from all SUVlean measurements for all tissues (*p* < 0.001). Another ANOVA test among SUV_dixon, SUV_james, and SUV_janma also showed significant difference (*p* < 0.001), except for SUVmax of the brain where *p* = 0.052.

### SUV in PSMA study

The SUVbw’s correlation with BMI was stronger than SUVlean for all tissues (Table 4). However, the correlation was significant only in the blood pool (*p* = 0.04) while not statistically significant within other organs. The dependency on BMI was eliminated after being corrected using the Dixon, James, or Janma-based SUVlean calculations as demonstrated by the reduced slope in supplementary material. One-way ANOVA showed significant difference between SUVlean measured using the Dixon method vs James and Janma models within normal organs. Quantitative statistics results are summarized in Table 4 and linear regression of PSMA SUVbw and SUVlean as a function of BMI is shown in supplementary material.

**Table 4:** Fitting results and dependent analysis of SUV vs BMI for the PSMA study (*n* = 28)

			Mean ± SD	CV	Slope with BMI	Correlation with BMI	Significance of correlation with BMI (*p*)	ANOVA
								(*p*)
Liver	Max	bw	8.85 ± 2.40	0.271	0.167	0.189	0.336	*p* < 0.001
		Dixon	5.97 ± 1.45	0.243	0.024	0.044	0.822	0.058
		James	6.96 ± 1.81	0.260	0.022	0.033	0.866	
		Janma	6.87 ± 1.77	0.258	0.010	0.016	0.938	
	Mean	bw	7.06 ± 2.04	0.289	0.148	0.197	0.316	*p* < 0.001
		Dixon	4.75 ± 1.24	0.260	0.032	0.070	0.725	0.080
		James	5.55 ± 1.55	0.279	0.032	0.056	0.776	
		Janma	5.47 ± 1.52	0.277	0.023	0.041	0.837	
Parotid	Max	bw	19.90 ± 5.62	0.283	0.208	0.101	0.611	*p* < 0.001
		Dixon	13.51 ± 3.93	0.291	0.004	0.003	0.990	0.120
		James	15.66 ± 4.44	0.283	− 0.054	− 0.033	0.867	
		Janma	15.45 ± 4.37	0.283	− 0.076	− 0.047	0.812	
	Mean	bw	10.32 ± 2.72	0.264	− 0.054	− 0.053	0.787	*p* < 0.001
		Dixon	7.02 ± 1.95	0.278	− 0.113	− 0.158	0.422	0.101
		James	8.13 ± 2.22	0.273	− 0.157	− 0.192	0.329	
		Janma	8.03 ± 2.19	0.273	− 0.166	− 0.206	0.293	
Blood	Max	bw	0.72 ± 0.23	0.325	0.033	0.389	0.041	*p* < 0.001
		Dixon	0.49 ± 0.15	0.318	0.017	0.298	0.124	0.166
		James	0.56 ± 0.17	0.302	0.017	0.267	0.169	
		Janma	0.56 ± 0.17	0.300	0.016	0.252	0.195	
	Mean	bw	0.54 ± 0.20	0.368	0.016	0.218	0.265	0.001
		Dixon	0.37 ± 0.14	0.371	0.006	0.129	0.514	0.266
		James	0.42 ± 0.15	0.360	0.006	0.104	0.600	
		Janma	0.42 ± 0.15	0.359	0.005	0.092	0.644	
Muscle	Max	bw	0.52 ± 0.18	0.351	0.009	0.129	0.513	*p* < 0.001
		Dixon	0.35 ± 0.12	0.334	0.001	0.031	0.878	0.198
		James	0.41 ± 0.14	0.347	0.001	0.009	0.964	
		Janma	0.40 ± 0.14	0.346	0.000	− 0.004	0.983	
	Mean	bw	0.33 ± 0.09	0.280	0.010	0.293	0.130	*p* < 0.001
		Dixon	0.22 ± 0.05	0.249	0.004	0.201	0.304	0.064
		James	0.26 ± 0.07	0.266	0.004	0.149	0.449	
		Janma	0.25 ± 0.07	0.265	0.003	0.133	0.501	
Prostate	Max	bw	14.0 ± 10.92	0.777	0.483	0.120	0.542	0.267
		Dixon	9.39 ± 7.20	0.767	0.200	0.075	0.703	0.709
		James	11.03 ± 8.71	0.790	0.200	0.062	0.753	
		Janma	10.88 ± 8.61	0.791	0.179	0.056	0.776	
	Mean	bw	5.89 ± 3.77	0.639	0.190	0.137	0.487	0.143
		Dixon	3.99 ± 2.67	0.670	0.074	0.075	0.705	0.660
		James	4.63 ± 3.00	0.648	0.073	0.066	0.738	
		Janma	4.56 ± 2.96	0.649	0.064	0.059	0.768	
Lesion	Peak	bw	4.53 ± 2.33	0.516	0.590	0.629	0.003	0.080
		Dixon	3.05 ± 1.55	0.507	0.357	0.575	0.008	0.655
		James	3.49 ± 1.72	0.493	0.403	0.583	0.007	
		Janma	3.44 ± 1.69	0.491	0.392	0.579	0.007	
	Mean	bw	3.86 ± 1.73	0.448	0.463	0.666	0.001	0.032
		Dixon	2.60 ± 1.15	0.443	0.278	0.600	0.005	0.565
		James	2.98 ± 1.28	0.428	0.312	0.609	0.004	
		Janma	2.94 ± 1.25	0.426	0.304	0.604	0.005	

*SUV*, standardized uptake value; *BMI*, body mass index; *CV*, coefficient of variation; *DXA*, dual energy X-ray absorptiometry; *SD*, standard deviation; *ANOVA*, analysis of variance

### Liver SUV among four BMI subgroups

**Fig. 5:**
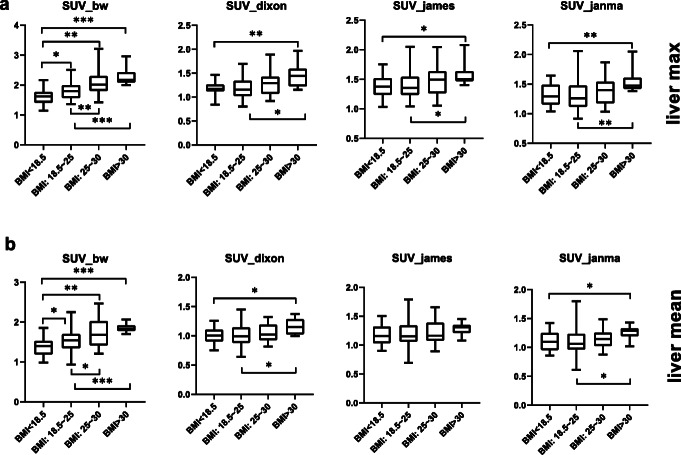
Box plot showing the liver SUV distribution of underweight (BMI < 18.5), normal (BMI 18.5–25), overweight (BMI 25–30), and obese (BMI > 30) patients. *** denotes *p* < 0.001 in an unpaired *t* test, ** denotes *p* < 0.01, * denotes *p* < 0.05

To further investigate whether obesity has an impact on SUV, we separated the 90 patients in the FDG study into four sub-groups according to BMI [34]: BMI < 18.5 were in the underweight group (*n* = 13), 18.5 < BMI < 25 were in the normal group (*n* = 50), 25 < BMI < 30 were in the overweight group (*n*=18), and BMI > 30 were in the obese group (*n* = 9). As demonstrated in Fig. 5, patients with larger BMIs have higher liver SUVbw and the difference is statistically significant. This positive correlation was mostly eliminated after LBM normalization.

## Discussion

Quantitation of tracer uptake from PET/MR images is essential for cancer staging and treatment response evaluation in both clinical and research settings. The pitfall of the widely used SUVbw is that it is highly dependent on patient weight and may overestimate the uptake in obese patients [5]. It has been reported that SUVbw was 70% higher in high-weight patients than in low-weight patients, and the overestimation was reduced when using other SUV normalization factors [35]. Using LBM instead of full body weight can effectively eliminate this effect and improve consistency among patients. Our findings in the FDG study were consistent to those reported by Zasadny [5] and Wahl [8, 12]. Our findings in the PSMA study were also in line with previously reported results [36, 37]. However, it is notable that, even though the multiple approaches all achieved satisfactory correction for the BMI dependency of SUV, significant differences were found among the Dixon, James, and Janma approaches.

Using DXA as the reference standard, this study compared, for the first time, the accuracy of these three widely used approaches to estimate LBM. All three methods were found to be in good agreement with DXA, with Dixon offering the smallest bias due to its direct measurement of body composition. The James and Janma models might be prone to individual bias due to the fact that BMI might not be fully indicative of body fat content, even though they both offer reasonable population-based estimates of body fat content in the recruited cohorts from our study.

The Dixon approach offers quantitative measurement of the water/fat volume from MRI images and has gradually established itself as an alternative LBM standard [23]. Jochimsen et al. [22] first proposed a method to correct SUV using the water/fat fraction from Dixon scans in 2015. In the present work, we revised their method to be more straight-forward and easy-to-implement and validated it using a larger patient cohort and an additional tracer. Our method is different from Jochimsen’s in that we utilized DXA measurements to transfer volume units into mass units, whereas they used signal intensity fraction instead.

It is notable that in previous reports, both body weight and BMI can be utilized as the dependent variable for SUV when evaluating the impact of obesity on SUV accuracy. We used BMI as the factor reflecting patient adiposity in our study because it is more indicative of actual fat content than body weight. For instance, patients from our cohort exhibited a wide range of body sizes, but the most obese subject, with the highest BMI, was of moderate bodyweight (80 kg) but very short in height (1.52 m). Therefore, weight may not be an ideal parameter to indicate the degree of obesity and we used BMI (BMI = weight/height^2^) as an indicator instead.

It is worthwhile mentioning that even though all three LBM approaches can be utilized to correct for the BMI dependency of SUV in FDG and PSMA studies, significant variation still exists among different approaches. A potential cause of this is that all empirical models including James and Janma are derived from specific patient populations while Dixon is a direct measurement of body composition. In serial PET/MR scans where quantitative accuracy is crucial, a consistent SUVlean calculation approach should be adopted, to minimize systematic bias, when correcting for the change of body weight and BMI index over time.

This study excluded patients with metal implants as they can induce severe imaging artifact in PET/MR [38], which could affect the image quality and qualitative results. Other imaging artifacts, such as motion, are minimized during the scan by asking the patients to breathe normally or hold breath during some of the MR sequences.

It is notable that statistically significant difference was found among SUV_lean calculations when using either the Dixon, James, or Janma methods in the FDG group. As the lean body weight derived from the Dixon images was found to be the most consistent with the DXA measurement, it indicates that the Dixon method might offer the most accurate correction factor for translating SUV_bw into SUV_lean for this particular patient cohort. However, Dixon is prone to multiple pitfalls caused by inherent limitations of the MRI acquisition (not full body) and image segmentation for the MRAC. One major challenge is that tissue characterization assumes uniform density for individual tissue type, but in real world clinical cases, such density might vary across different organs and patients. Both James and Janma equations were summarized based on large scale patient cohort in a western population; however, the validity of these empirical formulas for specific patient populations remains to be investigated.

There are a few limitations in this study. Firstly, since we were using 2-point Dixon which is the basic form of water fat imaging (WFI), we could only obtain the total amount of body fat by summing up the total number of categorized voxels. Further work might involve the use of more advanced WFI sequences (e.g., 6-point Dixon) that can differentiate different types of adipose tissue and derive the fat content within a single voxel. Brown adipose tissue that is typically active in glucose metabolism [39, 40] should not be subtracted from LBM. Secondly, although SUV dependency on BMI in the PSMA study was shown to be strong, the correlation between the SUV and BMI in most organs (except for blood pool) was not statistically significant. This could be due to the limited number of patients enrolled in this study. In a recent work by Grafita et al., a weak but significantly positive correlation was observed between liver SUV and body weight among 121 patients who underwent 68Ga-PSMA PET/CT. The SUVlean normalized to Janma LBM was reported to have a reduced correlation with body weight. Finally, the results of the lesion uptake were not in line with normal organs, possibly because lesions are heterogeneous in nature and subject to the specific biochemical differences between patients. The uptake of lesions depends mostly on characteristics of the tumor itself, such as tumor stage, size, degree of aggressiveness, and histology type.

## Conclusion

In this study, we have compared LBM calculated using the Dixon, James, and Janma approaches and validated their accuracy against DXA measurements. All three methods offer good estimates of LBM with the Dixon method offering the best agreement with DXA. SUVbw was found to be positively correlated with BMI in the FDG and PSMA patient populations while SUVlean calculated using Dixon, James and Janma methods confirmed a decreased dependence on BMI. However, significant systematic variation was found among SUVlean calculations using different approaches, suggesting that a consistent correction method would be needed for PET/MR serial scans.

## Supplementary Information

**Additional file 1:.** FDG

**Additional file 2:.** PSMA

## Data Availability

Not applicable.
