# Supplementary figures and images for: Evaluation of SUVlean consistency in FDG and PSMA PET/MR with Dixon-, James-, and Janma-based lean body mass correction
Source: EJNMMI Phys. 2021 Feb 17;8:17. doi: 10.1186/s40658-021-00363-w (PMC7889776; doi:10.1186/s40658-021-00363-w)

**SUV\_bw****SUV\_dixon****SUV\_james****SUV\_janma****a**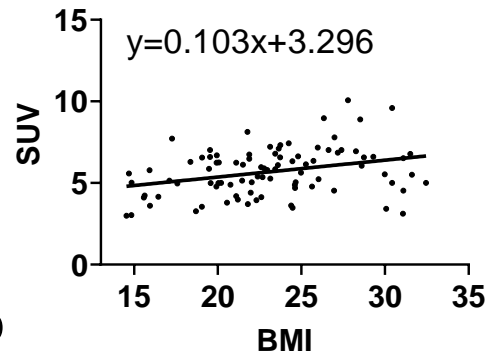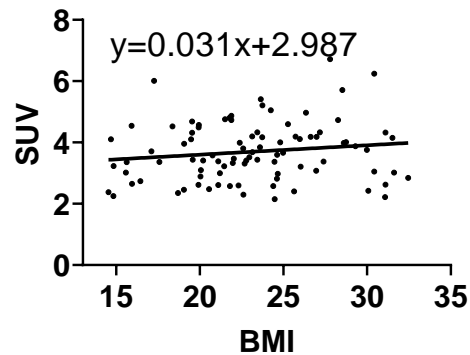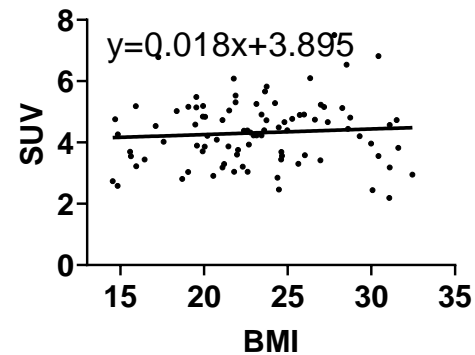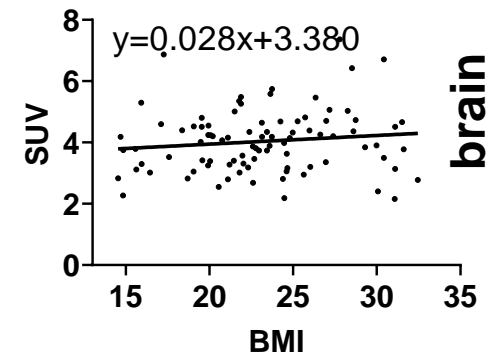**brain****b**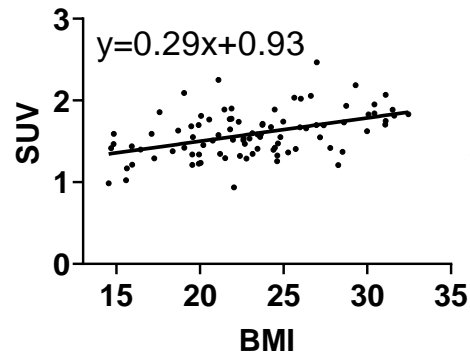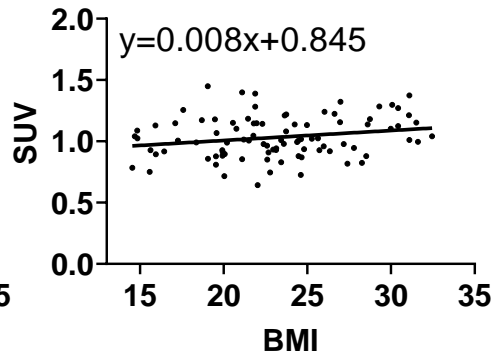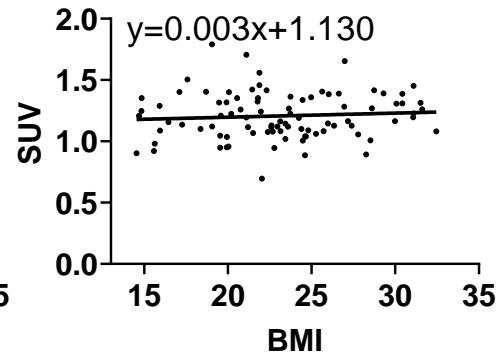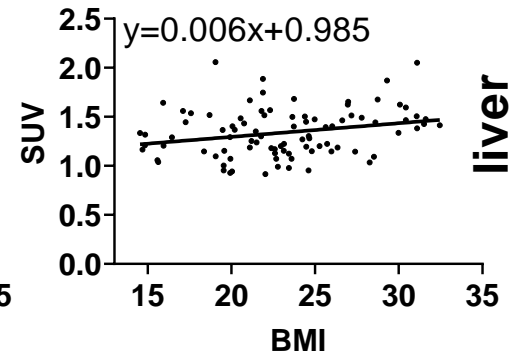**liver****c**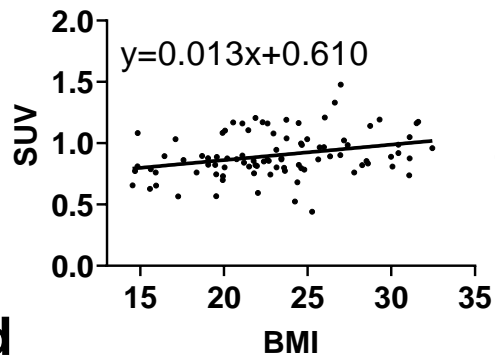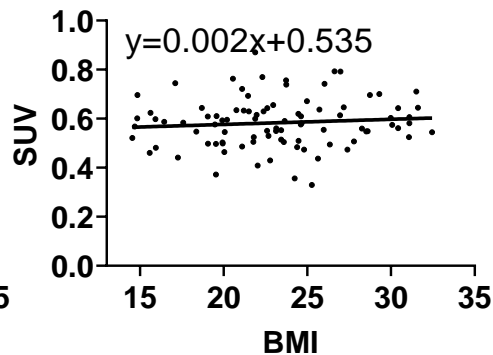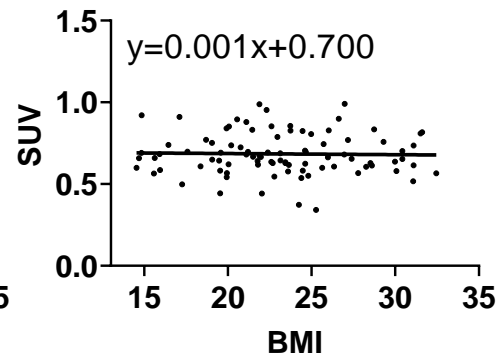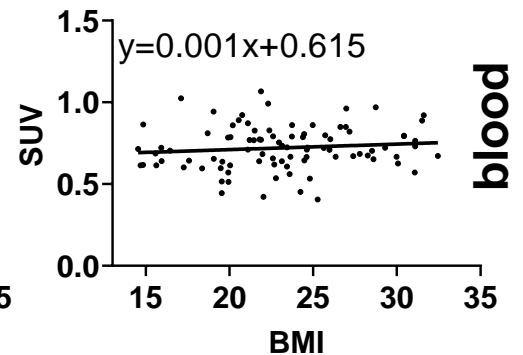**blood****d**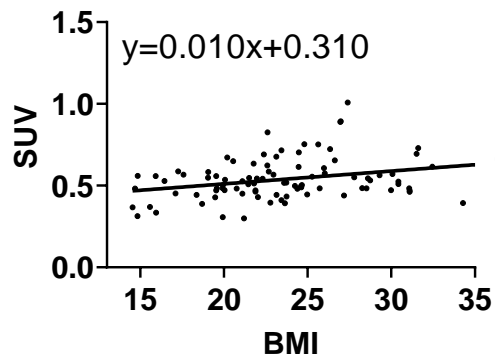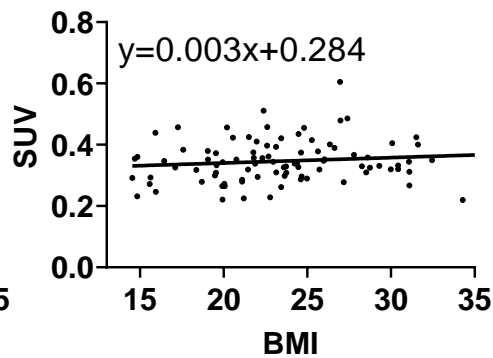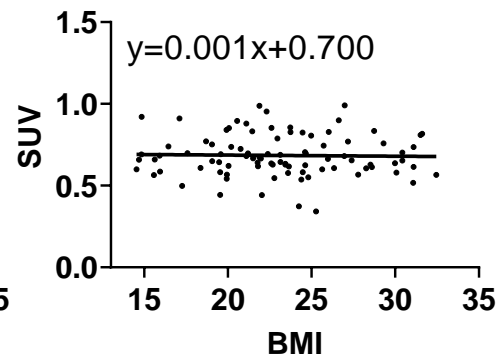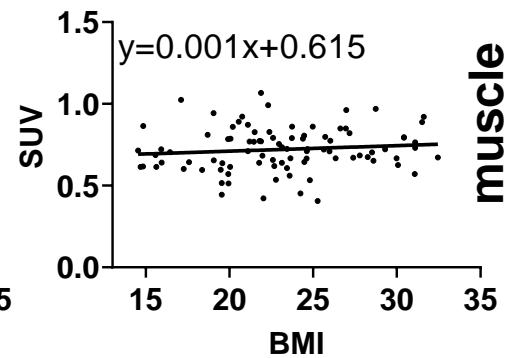**muscle**

Supplement: Supplementary file 1 — Additional file 1:. FDG [file 40658_2021_363_MOESM1_ESM.pdf]

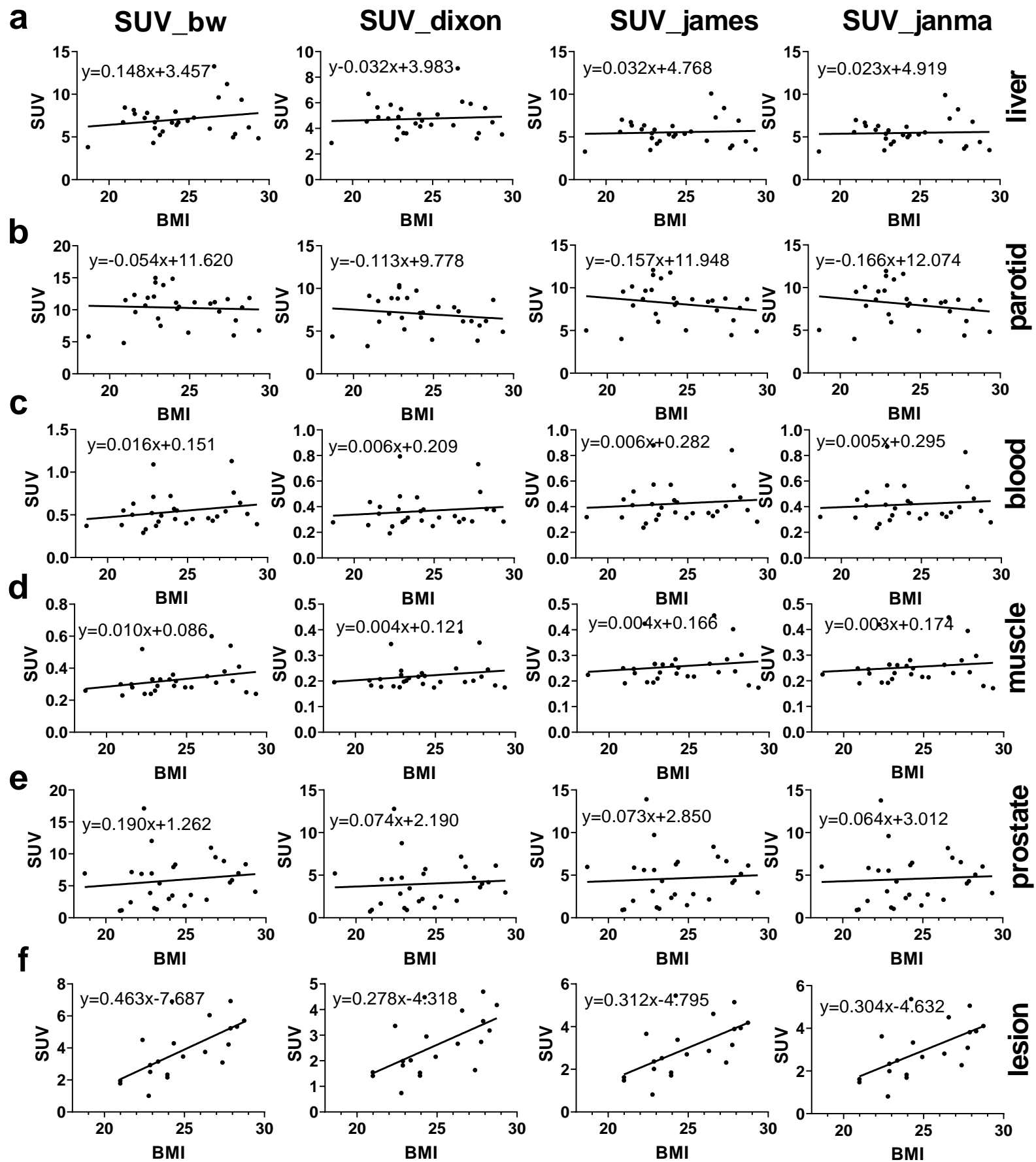

Supplement: Supplementary file 2 — Additional file 2:. PSMA [file 40658_2021_363_MOESM2_ESM.pdf]
